# Recent advances in cell membrane-camouflaged nanoparticles for inflammation therapy

**DOI:** 10.1080/10717544.2021.1934188

**Published:** 2021-06-12

**Authors:** Rongtao Zhang, Siqiong Wu, Qian Ding, Qingze Fan, Yan Dai, Shiwei Guo, Yun Ye, Chunhong Li, Meiling Zhou

**Affiliations:** a Department of Pharmacy, the Affiliated Hospital of Southwest Medical University, Luzhou, China; b School of Pharmacy, Southwest Medical University, Luzhou, China; c Department of Pharmaceutical Sciences, School of Pharmacy, Southwest Medical University, Luzhou, China

**Keywords:** Cell membrane, camouflaged, nanoparticles, inflammation, drug delivery

## Abstract

During inflammation, inflammatory cells are rapidly recruited to sites of infection or injury, where they cross physiological barriers around the infected site and further infiltrate into the tissues. Other cells, such as erythrocytes, endothelial cells and stem cells, also play prominent roles in host defense and tissue repair. In recent years, nanotechnology has been exploited to deliver drugs to sites of inflammation. For example, nanoparticles camouflaged with a cell membrane are a novel drug-delivery platform that can interact with the immune system and that show great potential for treating inflammation. Encapsulating drugs inside plasma membranes derived from various cells involved in inflammatory processes can be effective against inflammation. This review describes the preparation, characterization, and properties of various types of cell membrane-camouflaged biomimetic nanoparticles. It also summarizes preclinical research into their efficacy against inflammation.

## Introduction

Inflammation is the normal immune response against harmful factors and insults such as pathogens, injuries and irritants (Han, et al., 2018). During inflammation, immune cells such as neutrophils, monocytes and lymphocytes infiltrate into affected tissues to varying degrees. Acute inflammation is a necessary physiological process to prevent infection, eliminate damaging factors and contribute to the healing of damaged tissue (Sonnenberg & Artis, 2015). Chronic inflammation, however, can increase the risk of inflammatory damage as well as infectious and other systemic diseases (Nasef et al., 2017), including arthritis, atherosclerosis and cancer (Tabas & Glass, 2013; Dong et al., 2017).

Several classes of drugs can be effective against inflammation, including antibiotics, cytotoxic drugs and hormones, but they act systemically, so their long-term use can cause serious side effects (Hou et al., 2015; Arulselvan et al., 2016). A safer and more effective alternative may be to target anti-inflammatory drugs using nanoparticle-based drug delivery systems. For example, glucocorticoids have been encapsulated in long-circulating PEG liposomes for arthritis treatment (Metselaar et al., 2003), dexamethasone has been loaded into PEG-PLGA nanospheres to treat mesangial proliferative glomerulonephritis (Li et al., 2019), and silica nanoparticles coated with cerium dioxide have been used to treat pneumonia (Serebrovska et al., 2017). While nanoparticles can protect the body from a drug’s adverse effects, they can pose a problem of their own: the immune system may recognize the nanoparticles and clear them rapidly, or even launch an innate immune response that elicits toxic effects (Kononenko et al., 2015).

One solution to these problems is to formulate nanoparticles that simulate the function and structure of endogenous substances. These so-called ‘biomimetic’ drug delivery systems can escape being phagocytosed by the mononuclear phagocytic system (MPS), prolonging their circulation in the bloodstream and therefore drug delivery to the targeted site (Fang et al., 2012). A promising type of biomimetic drug delivery system is nanoparticles camouflaged with membranes derived from erythrocytes, platelets, leukocytes or macrophages. For example, nanoparticles camouflaged within leukocyte-derived membrane can target inflamed endothelium and transmigrate through the endothelial barrier while eluding lysosomal degradation (Parodi et al., 2013). As another example, nanoparticles camouflaged in membrane derived from the outer membrane of pathogenic bacteria, which displays pathogen associated-molecular patterns, can stimulate innate immunity and promote adaptive immune responses, giving them potential as antibacterial vaccines (Gao et al., 2015).

**Figure 1.**
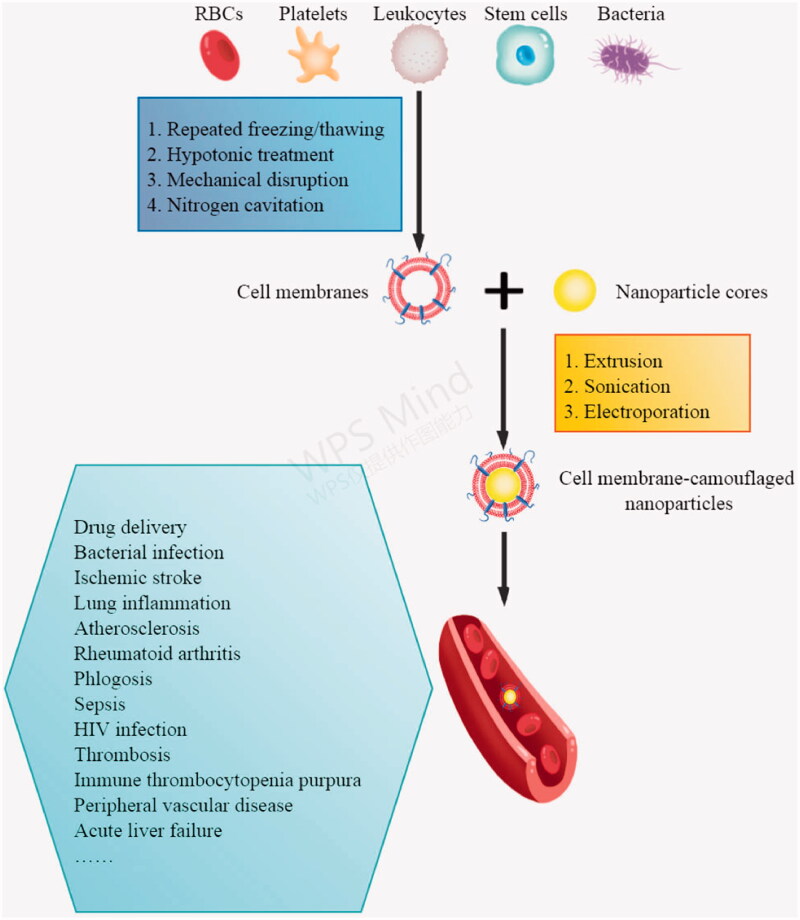
Schematic illustration of the preparation of cell membrane-camouflaged nanoparticles and their applications. RBCs, red blood cells.

The purpose of the present review is to summarize recent development in preparing cell membrane-camouflaged nanoparticles and using them to treat inflammation (Figure 1).

## Composition of cell membrane-camouflaged biomimetic nanoparticles

In cell membrane-camouflaged biomimetic nanoparticles, the nanoparticle lies at the core, while the cell membrane forms the outer shell. The core not only supports the membrane but it also contains cargo such as therapeutic agents and it can bear structural modifications. The membrane is freshly extracted from the appropriate cell types in a process designed to preserve the membrane’s biological and functional properties (Luk & Zhang, 2015). Then the biomimetic nanoparticles are assembled through extrusion, sonication or electroporation. The resulting nanoparticles appear as ‘self’ and ‘cell-like’ to the organism from which the membrane was derived (Tan et al., 2015). As a result, the nanoparticles can evade the immune system, persist in circulation, and be recruited to sites of inflammation.

Popular sources of cell membrane for encapsulating nanoparticles include red blood cells (RBCs) (Rossi et al., 2019; Yang et al., 2019), leukocytes (Huang et al., 2018), stem cells (Bose et al., 2018; Wu et al., 2019), platelets (Wei et al., 2016; Li et al., 2018), cancer cells (Harris et al., 2019) and bacteria (Zhang et al., 2019). The source is chosen in order to obtain a membrane that will confer the desired properties on the resulting camouflaged nanoparticles. For example, RBC membranes can avoid recognition by the MPS and prolong circulation time. Leukocyte membranes bind to endothelial cells at sites of inflammation, allowing them to be recruited (Molinaro et al., 2016). Membranes derived from bacterial outer membrane vesicles (OMVs) may migrate to sites of infection, where they can trigger specific immunity.

The nanoparticle core is chosen according to the physicochemical properties of the drug cargo. The most popular cores are poly(lactic-co-glycolic acid) (PLGA), poly(ε-caprolactone), gelatin, nanogel, liposome, porous silicon, gold nanoparticles and iron oxide nanoparticles (Hoshyar et al., 2016; Bobo et al., 2020).

When the camouflaged nanoparticles reach the target tissue, the outer membrane ruptures, releasing the drug cargo. The empty core is then degraded and excreted from the body.

## Preparation of cell membrane-camouflaged biomimetic nanoparticles

### Separation and extraction of cell membranes

Cell membranes and vesicles derived from them are asymmetric phospholipid bilayers that may contain thousands of unique membrane proteins that are essential for their biological functions (van Meer, 2011). Methods commonly used to fuze membranes include extrusion, ultrasonic fusion and electroporation. Recently, nitrogen cavitation has been used to disrupt cells and generate pure, membrane-bound nanovesicles (Gao et al., 2016, 2017). Subsequent differential centrifugation can purify the nanovesicles away from other intracellular contents of the parent cells (Wang et al., 2018). In order to minimize the denaturation of membrane proteins, the extraction and purification of cell membranes must be performed under as mild conditions as possible. To maintain their biological activity, the prepared cell membranes should be used immediately, or they may be aliquoted and stored at −80 °C, sometimes in the presence of protease inhibitors to prevent membrane protein degradation (Gao et al., 2015; Hu et al., 2015). The precise extraction approach depends on whether the source cell contains a nucleus.

#### Extraction of membranes from enucleated or bacterial cells

Erythrocytes and platelets, which are highly differentiated enucleated cells, are isolated from whole blood using a blood separation kit or centrifugation, then subjected to repeated freezing/thawing, hypotonic cycles or nitrogen cavitation, during which soluble proteins are removed by differential centrifugation. In order to avoid platelet activation, pure platelets are prepared, usually in the presence of EDTA (Hu et al., 2015; Wang et al., 2019). To maintain the biological activity of the membrane, protease inhibitors are usually added to the samples, which are stored at 4 °C (Zhai et al., 2017; Xia et al., 2019). Extruding the crude membranes repeatedly through polycarbonate membranes or ultrasonication affords nano-scale cell membrane fragments or vesicles (Tan et al., 2015; Xia et al., 2019).

Gram-negative bacteria, which also lack a nucleus, generally have a three-layer structure: an outer membrane covers the peptidoglycan periplasm, which in turn covers the lipid inner membrane (Fisher & Mobashery, 2020). Bacterial OMVs can be extracted using buffer solution or nitrogen cavitation, and then cell debris is removed by low-speed centrifugation or filtration (Holst et al., 2009). OMVs can also be harvested directly from culture medium after filtering out the bacteria (Gao et al., 2015).

#### Extraction of membranes from eukaryotic cells

It is more challenging to extract and purify cell membranes from eukaryotic cells than from enucleated or bacterial cells. First, a sufficient number of source cells are collected from blood, from tissue samples or in the case of stem cells or cancer cells – from cultures (Fang et al., 2014; Gao et al., 2016). Then, the cells are lysed via repeated freezing and thawing, hypotonic treatment, mechanical disruption or nitrogen cavitation. Unbroken cells, nuclei and other subcellular components are removed by gradient centrifugation, and the cell membranes are further purified (Cao et al., 2016; Oieni et al., 2020). The purified membranes are extruded through polycarbonate membranes or ultrasonicated to form membrane fragments or vesicles.

### Preparation of cell membrane-camouflaged nanoparticles

Cell membrane-camouflaged nanoparticles are prepared by fuzing membrane fragments or vesicles with nanoparticles. Care should be taken to ensure that membranes remain intact throughout the fusion step, in order to avoid drug leakage. The three methods most often used to prepare cell membrane-camouflaged nanoparticles are extrusion, sonication and electroporation (Xia et al., 2019; Choi et al., 2020).

#### Extrusion

The mixture of cell membranes and nanoparticles is repeatedly extruded successively through polycarbonate membranes with pore diameters of 1000, 800, 400, 200 and 100 nm at least 5 times in order to form particles of the expected size (Ren et al., 2016; Rao et al., 2020). Excess membrane is usually used in order to ensure complete coating of nanoparticles despite membrane losses during the fusion process. This method can be used to encapsulate polymeric nanoparticles with sizes ranging from 65 to 340 nm (Xia et al., 2019). The biomimetic nanoparticles prepared in this way show uniform size and encapsulate drugs efficiently. However, this method is time-consuming and costly for large-scale production (Guo et al., 2018).

#### Sonication

Sonication can induce nanoparticle cores to fuze with cell membranes via electrostatic interactions. One group prepared platelet membrane-coated nanoparticles via sonication using a bath sonicator at a frequency of 42 kHz and a power of 100 W for 2 minutes (Wei et al., 2016). While this procedure is simple, it easily results in a heterogeneous distribution of membranes, leading to polydisperse preparations (Vijayan et al., 2018). Ultrasonication frequency, power and duration should be optimized to maximize fusion efficiency as well as minimize degradation or denaturation of membrane proteins and drug leakage.

#### Electroporation

During electroporation, application of an external electric field creates many transient pores in the cell membrane, increasing its permeability and allowing the entry of nanoparticle cores or drug molecules (Shi et al., 2018). The specific response varies with cell size, membrane composition, electric field strength and other experimental conditions (Krassowska & Filev, 2007). As the intensity of the pulse increases, so does the number of small pores. Erythrocyte membrane-coated nanoparticles with a diameter of approximately 100 nm can be synthesized when the pulse voltage is 50 V, pulse duration is 200 μs, and flow velocity is 20 μL/min (Rao et al., 2017). The amplitude and duration of the electric field application should be optimized to avoid irreversible damage to the cell membrane (Tan et al., 2015; Rao et al., 2017). It is possible that unwanted contaminants can enter the membranes together with the desired drug cargo during electroporation, and that these contaminants promote phagocytosis of the phagocytic cells in the body, thereby reducing the circulation time of biomimetic nanoparticles (Tarek, 2017).

Regardless of which of these three preparation methods is used, the ratio of membranes to nanoparticle cores should be optimized to ensure complete camouflaging of the core’s surface (Hu et al., 2013; Xia et al., 2019).

## Characterization of cell membrane-camouflaged biomimetic nanoparticles

### Physicochemical properties

**Figure 2.**
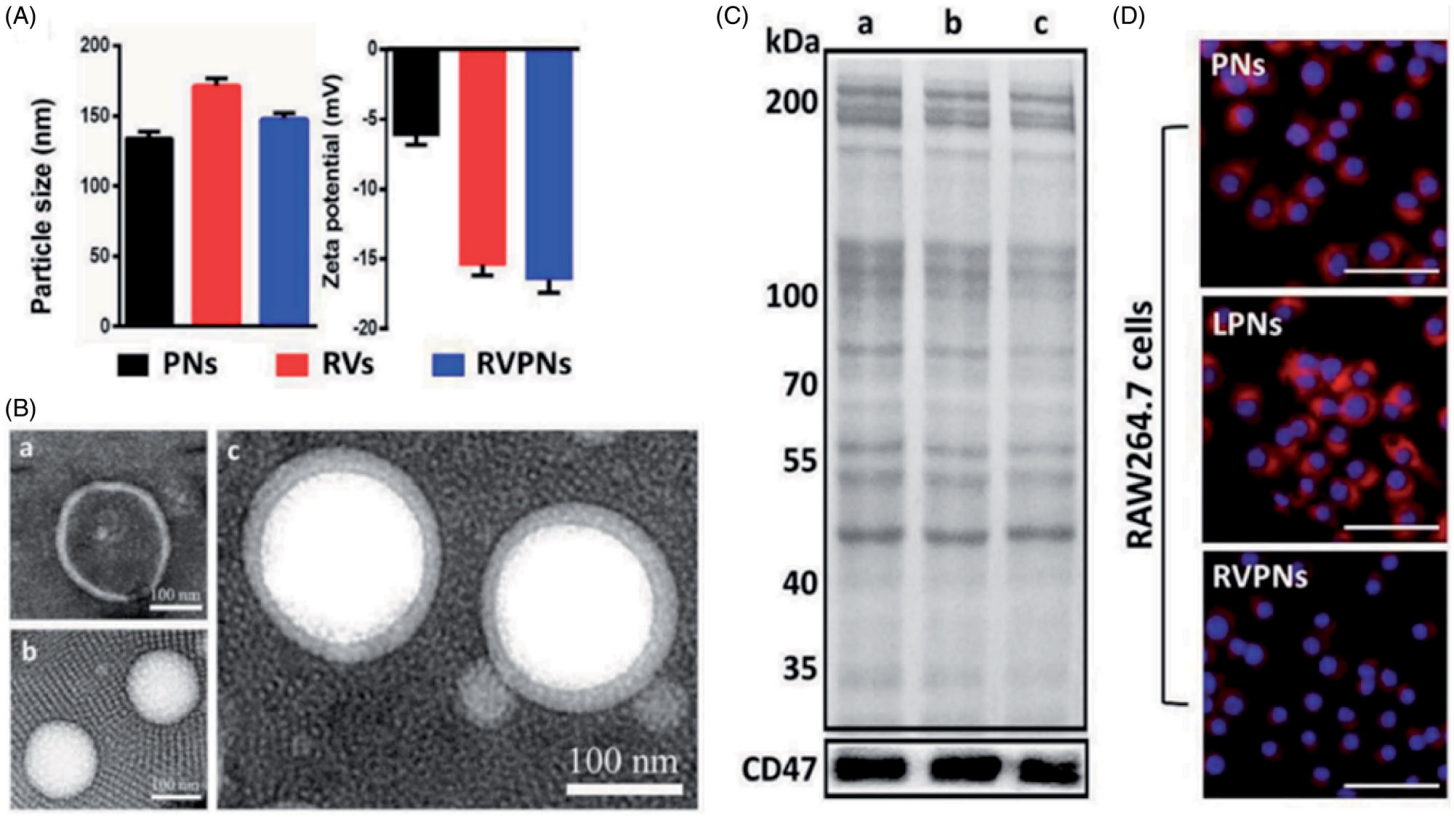
Biophysical characterization of RBC-mimicking PLGA nanoparticles. (A) Particle size and zeta potential. (B) Representative TEM images. (C) Presence of membrane proteins in the nanoparticles, based on total SDS-PAGE analysis (*upper*) and western blot analysis against CD47 (*lower*). (D) Uptake of RBC-mimicking PLGA nanoparticles by macrophages. DAPI-stained nuclei appear blue, while NR-labeled nanoparticles appear red. PNs: hybrid polymeric nanoparticles, RVs: red blood cell vesicles, RVPNs: RV-coated PNs, LPNs: PNs coated with artificial lipid membrane (Su et al., 2016). Copyright 2016, John Wiley and Sons.

The size, polydispersity index (PDI) and zeta potential of the camouflaged nanoparticles are determined using dynamic light scattering (Luk et al., 2014). The cell membrane coating usually alters the electrical potential and size of the nanoparticle core, thereby significantly improving their colloidal stability (Gao et al. 2016; Zhang et al., 2019). For instance, PLGA nanoparticle cores have a diameter of 133.9 nm, which increases to 147.9 nm after fusion with RBC membrane (Figure 2(A)). These camouflaged nanoparticles have a zeta potential of −16.1 mV, comparable to the −15.2 mV of RBC vesicles, indicating successful membrane coating of the nanoparticle core (Su et al., 2016).

Membrane encapsulation of nanoparticle cores is confirmed by observing the ‘core-shell’ structure by transmission electron microscopy (TEM) (Hu et al., 2011). Because of their different electron densities, naked nanoparticle cores appear as nearly white spheres, disrupted membranes appear light gray, and intact membrane-camouflaged nanoparticles show both elements within a ‘core-shell’ structure (Su et al., 2016) (Figure 2(B)).

### Biological properties

Analysis of physicochemical properties of camouflaged nanoparticles can reveal whether or not the core is fully encapsulated within the membrane shell. However, additional analysis is needed in order to determine whether the membrane retains the proteins from the donor cell type (Fang et al., 2014; Cao et al., 2016). Sodium dodecyl sulfate-polyacrylamide gel electrophoresis can be used to compare the protein content of the purified membranes with the proteins in the donor cell type (Figure 2(C)), while western blotting can confirm the presence of desired proteins.

## Preclinical studies of cell membrane-camouflaged biomimetic nanoparticles against inflammation

Cell membrane-camouflaged biomimetic nanoparticles show the potential to deliver drugs specifically to sites of inflammation, thereby avoiding the systemic toxicity that often occurs with current treatments for chronic inflammation (Table 1). In these formulations, nanoparticle cores are encapsulated in different types of membranes in order to achieve different therapeutic functions.

**Table 1.** Preclinical studies of cell membrane-camouflaged biomimetic nanoparticles against inflammation.

Source of membrane	Nanoparticle core	Cargo inside core	Application/treatment	Ref.
Erythrocytes	PLGA	Staphylococcal α-haemolysin	Antitoxin vaccine	(Hu et al., 2013)
	Supramolecular gelatin	Vancomycin	Bacterial infection	(Li et al., 2014)
	–	IL-1β and ceftriaxone	Purulent inflammation	(Berikkhanova et al., 2016)
	Dextran polymer core	Neuroprotective agent NR2B9C	Ischemic stroke	(Lv et al., 2018)
	–	Betamethasone phosphate sodium	Long-term anti-inflammation	(Zhang et al., 2018)
	PLGA	–	Group B *Streptococcus* infection	(Koo et al., 2019)
	NP/pZNF580 complexes	–	Drug delivery	(Hao et al., 2018)
	Nanogel	Antibiotic	*Staphylococcus aureus* infection	(Zhang et al., 2017)
	PLGA	Rapamycin	Atherosclerosis	(Wang et al., 2019)
Leukocytes	Nanoporous silicon	–	Drug delivery	(Parodi et al., 2013)
	Lipid nanoparticles	Dexamethasone	Phlogosis	(Molinaro et al., 2016)
	Leukosome	Rapamycin	Atherosclerosis	(Boada et al., 2019)
Neutrophils	PLGA	–	Rheumatoid arthritis	(Zhang et al. 2018)
Macrophages	PLGA	–	Sepsis	(Thamphiwatana et al., 2017)
	Gold-silver nanocage	–	Antibacterial	(Wang et al., 2018)
Lymphocytes	PLGA	–	HIV infection	(Wei et al., 2018)
Platelets	PLGA	–	Thrombosis	(Doshi et al., 2012)
	PLGA	Docetaxel/Vancomycin	Coronary restenosis /Systemic bacterial infection	(Hu et al. 2015)
	PLGA	–	Immune thrombocytopenia purpura	(Wei et al., 2016)
	PLGA	FK506	Rheumatoid arthritis	(He et al., 2018)
	PLGA	Rapamycin	Atherosclerosis	(Song et al., 2019)
Human adipose stem cells	PLGA	Vascular endothelial growth factor	Peripheral vascular disease	(Bose et al., 2018)
Bacterial	Au nanoparticles	–	Antibacterial vaccine	(Gao et al., 2015)
	eMMT-lPEI nanoparticles	Metronidazole	*Helicobacter pylori* infection	(Ping et al., 2016)
	PLGA	–	*Helicobacter pylori* infection	(Zhang et al., 2019)
Erythrocyte-platelet	PLGA	–	Drug delivery	(Dehaini et al., 2017)
	acoustic gold nanowires	–	Bacterial infection	(Esteban-Fernández de Ávila et al., 2018)
BMSCs-erythrocytes	PLGA	–	Acute liver failure	(Liang et al., 2018)
Gastric epithelial cell	PLGA	Clarithromycin	*Helicobacter pylori* infection	(Angsantikul et al., 2018)

*Abbreviations.* PLGA: poly(lactic-co-glycolic acid); NP/pZNF580 complexes: amphiphilic polymer complexed with plasmid (pZNF580); FK506: a potent immunosuppressive agent used for rheumatoid arthritis therapy; eMMT-1PEI: highly exfoliated ultrathin montmorillonite nanosheet.

### Biomimetic nanoparticles camouflaged with erythrocyte membrane

Erythrocytes are the most numerous and long-lived cells in the blood, circulating for up to 120 days. They can be administered intravascularly and show excellent biocompatibility, complete degradation and no immunogenicity (Muzykantov, 2010; Hu et al., 2012). Coating nanoparticles with erythrocyte membrane allows them to escape the immune system and circulate for a long time in the blood (Figure 2(D)). Both characteristics are essential for drug delivery.

Encapsulating IL-1β and ceftriaxone into nanoparticles camouflaged with RBC membrane prolonged the half-life of IL-1β in the blood and promoted its distribution to the liver, spleen and lung (Berikkhanova et al., 2016). Encapsulating betamethasone phosphate directly into erythrocytes allowed the drug to circulate in the blood for longer than 9 days, it was released for longer than 7 days, and it exerted anti-inflammatory effects *in vivo* for at least 5 days (Vijayan et al., 2018).

**Figure 3.**
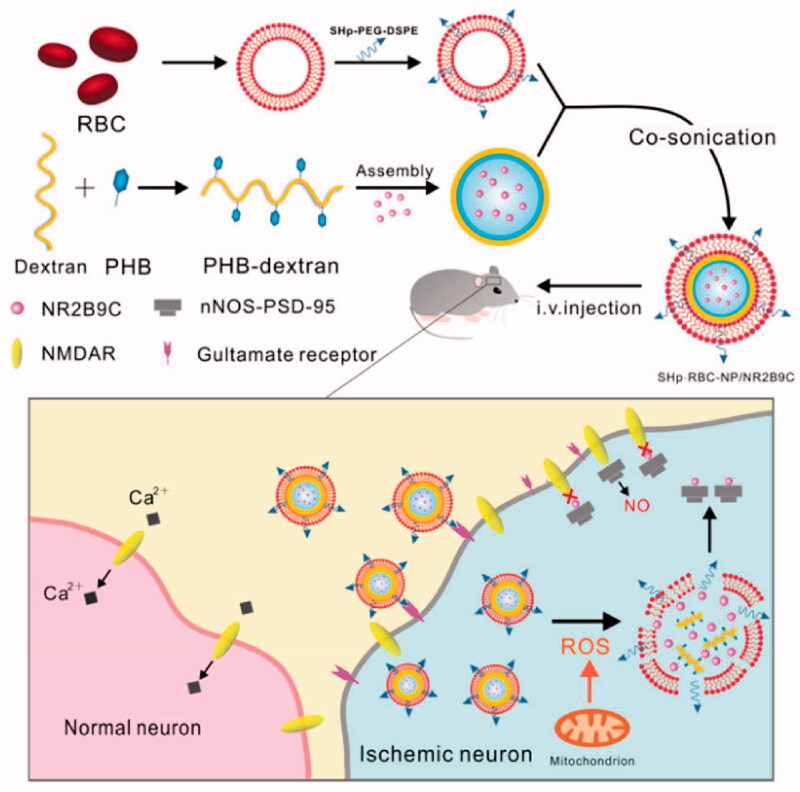
Schematic of the preparation and mechanism of action of nanoparticles camouflaged with RBC membrane that contains the stroke homing peptide. The nanoparticles are loaded with the neuroprotective agent NR2B9C. PEG, polyethylene glycol; DSPE, 1, 2-distearoyl-*sn*-glycero-3-phosphoethanolamine; PHB, poly-β-hydroxybutyrate; RBC, red blood cells; ROS, reactive oxygen species; NO, nitric oxide; SHp, stroke-homing peptide (Lv et al., 2018). Copyright 2018, American Chemical Society.

Nanoparticles camouflaged with RBC membrane can be targeted by introducing appropriate ligands into the membrane. For example, nanoparticles that respond to reactive oxygen species have been coated with stroke homing peptide in the membrane and loaded with the neuroprotective agent NR2B9C (Lv et al., 2018). The homing peptide leads the nanoparticles to be recruited to sites of ischemia (Figure 3).

### Biomimetic nanoparticles camouflaged with leukocyte membrane

Leukocytes comprise several subtypes, including neutrophils, macrophages, monocytes and lymphocytes. They are part of the innate immune system and play an important role in inflammatory processes, even helping to keep them under control. For example, neutrophils and macrophages can neutralize the toxic effects of endotoxin and proinflammatory cytokines, alleviating the symptoms of septicemia and arthritis. Although leukocytes circulate in the blood for a shorter time than erythrocytes (up to 20 days), they can cross physiological barriers to permeate tissue. Their surface receptors can bind to ligands on endothelium, allowing them to migrate to sites of inflammation (Mitchell & King, 2015; Molinaro et al., 2016). Thus, leukocyte membranes can target nanoparticles to sites of inflammation, where they efficiently release drugs.

#### Nanoparticles coated with neutrophil membrane

In one study, porous silicon nanoparticles were coated with leukocyte membranes, which were confirmed to retain major proteins of the donor cells, including those important for interaction with lymphocytes and adhesion to epithelial cells (Parodi et al., 2013). These camouflaged nanoparticles avoided uptake by the MPS or opsonization, they interacted with inflammatory endothelial cells, and they facilitated drug transport across the endothelium while eluding lysosomal degradation.

**Figure 4.**
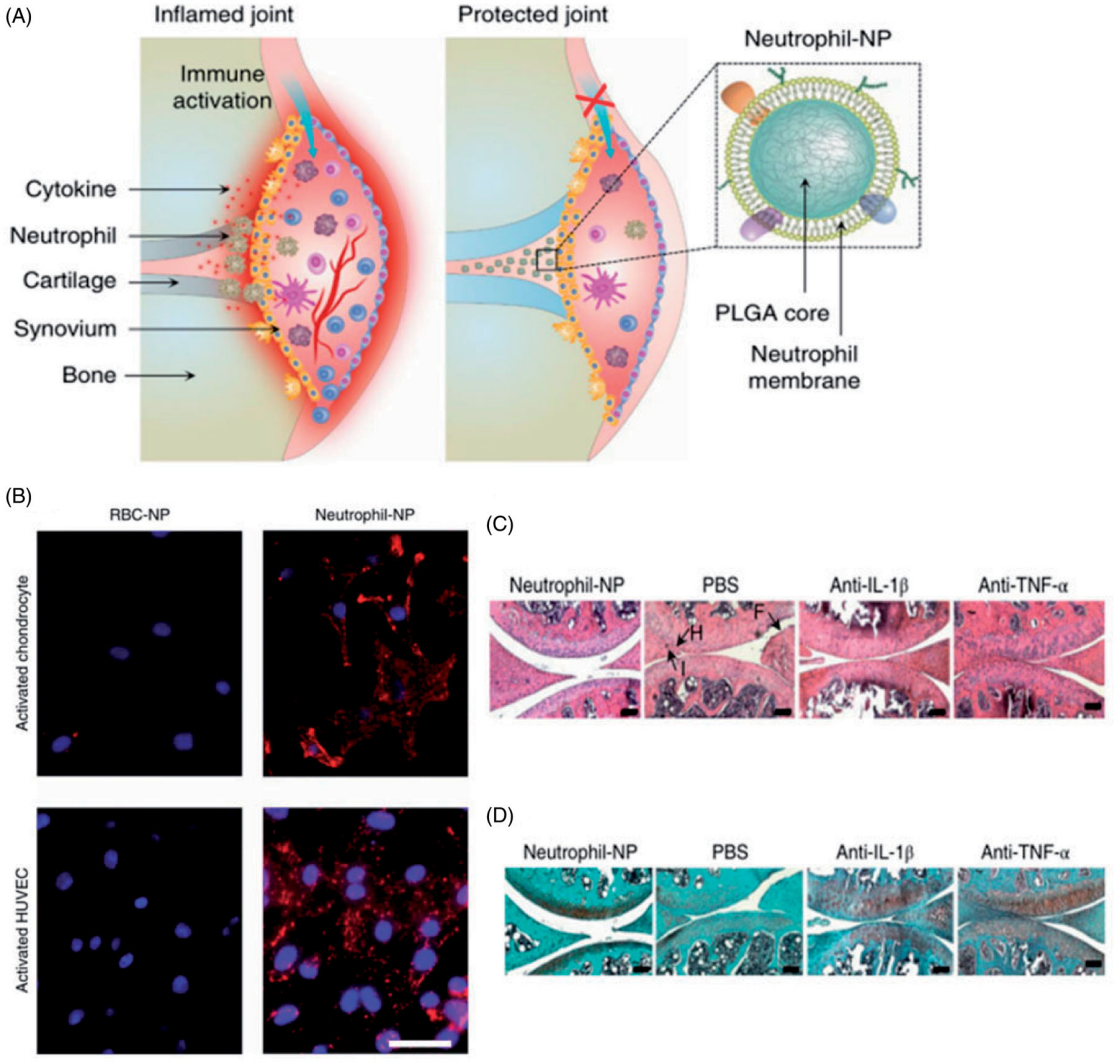
(A) Schematic representation of neutrophil membrane-camouflaged nanoparticles (NPs) for suppressing synovial inflammation and ameliorating joint destruction in inflammatory arthritis. (B) Fluorescent images of chondrocytes and human umbilical vein endothelial cells (HUVECs) after incubation with neutrophil-NPs or red blood cell (RBC)-NPs. Nuclei appear blue; nanoparticles, red. Scale bar, 50 μm. (C–D) Representative micrographs of knee sections from mice treated with neutrophil-NPs or phosphate-buffered saline (PBS), then stained with (C) H&E staining or (D) safranin-O. Some sections were also immunostained with antibody against IL-1β or TNF-α. Scale bars, 100 μm. F, synovial membrane fibrillation; H, synovium hyperplasia; I, immune cell infiltration; RBC-NP, nanoparticle coated with red blood cell membrane; neutrophil-NP, nanoparticle coated with neutrophil membrane; PLGA, poly(lactic-co-glycolic acid) (Zhang et al., 2018). Copyright 2018, Springer Nature.

Neutrophils are the most abundant white blood cells in the innate immune system. Neutrophils are not restricted to a particular part of the circulation: they can move freely through vein walls and tissues to attack antigens immediately (Kolaczkowska & Kubes, 2013). Neutrophils act through their membrane to detect cytokines and chemokines, which recruit them to sites of inflammation; neutrophils also aggregate with each other through their membranes in order to exert anti-inflammatory effects (Kolaczkowska & Kubes, 2013). In a mouse model, coating nanoparticle cores with neutrophil membrane (Figure 4(A)) allowed them to neutralize pro-inflammatory cytokines, suppress synovial inflammation, target deep into the cartilage matrix and provide strong chondroprotection against joint damage (Zhang et al., 2018) (Figure 4(B)). Histology showed normal articular cartilage and significantly less neutrophil infiltration in tissue treated with the nanoparticles than in control tissue, which showed nearly no chondrocytes and obvious degeneration of joints and synovium (Figure 4(C, D)).

Nanovesicles from neutrophils can specifically target inflamed endothelium in brain and lungs in order to treat stroke and acute lung inflammation (Dong et al., 2019; Gao et al., 2020). Nitrogen cavitation can rapidly disrupt cells to form pure nanovesicles from cell membrane (Gao et al. 2016). In one study, incorporating resolvin D1 into the lipid bilayer of neutrophil membrane allowed such nanovesicles to adhere specifically to endothelial cells for the treatment of ischemic stroke lesions and lung infection (Gao et al., 2021). Resolvin D1 blocks infiltration by neutrophils and promotes the clearance of macrophages, helping to resolve inflammation (Gao et al., 2020, 2021). These studies demonstrate that neutrophil membrane nanovesicles possess significant therapeutic potential for the treatment of inflammatory disorders.

#### Nanoparticles coated with macrophage membrane

Macrophages are white blood cells that can recognize, phagocytose and digest cell debris and foreign substances. Similar to neutrophils, they play a crucial role in inflammation and vascular injury. They also recruit other immune cells to sites of infection, they phagocytose pathogens and they activate complement and the adaptive immune system. Macrophages secrete cytokines and chemokines that drive tissue healing after injury (Sridharan et al., 2015; Oishi & Manabe, 2018).

Nanoparticle cores wrapped with membrane derived from mononuclear macrophages can be used for imaging brain tumors and targeting inflammation (Hwang et al., 2015). Polymeric cores wrapped with macrophage membrane can bind and neutralize endotoxins through homologous pattern recognition receptors such as TLR4 and CD14 (Thamphiwatana et al., 2017). The biomimetic nanoparticles also contain surface receptors CD126, CD130, CD120a/b and CD119, allowing them to bind pro-inflammatory cytokines and thereby inhibit the sepsis cascade. Macrophage-mimicking nanoparticles have been shown to reduce levels of pro-inflammatory cytokines and improve survival in *Escherichia coli* infection. Since macrophage membranes carry bacterial recognition receptors that can trigger responses to infection, extracting such membranes and using them to wrap nanoparticle cores can result in bacteria-targeting particles. In one study comparing these wrapped cores to naked nanoparticle cores, the wrapped cores were able to bind to *Staphylococcus aureus* or *Escherichia coli* much more, they persisted longer in the circulation and they accumulated to a greater extent at sites of inflammation (Wang et al., 2018).

#### Nanoparticles coated with lymphocyte membrane

Cytotoxic T lymphocytes (CTLs) kill cancer cells and other infected cells. CTLs can promote target cell death (apoptosis) through a granule- and receptor-mediated mechanism (Qin et al., 2016). The chemokine receptors CCR5 and CXCR4 on the CTL surface are recognized by viral fusion proteins (Campbell & Hope, 2015), which inspired researchers to wrap CTL membranes around polymer nanoparticles, generating a system that could selectively bind gp120 on the surface of the human immunodeficiency virus (Wei et al., 2018).

### Nanoparticles coated with platelet membrane and their anti-inflammatory activity

Platelets are enucleated blood cells that are derived from megakaryocytes. Platelets are major actors in hemostasis, thrombosis, inflammation as well as host and adaptive immune responses (Anitua et al., 2004; Etulain, 2018). Many inflammatory diseases involve platelet activation, including cardiovascular disease, sepsis, inflammatory bowel disease, arthritis and cancer (May et al., 2008; De Stoppelaar et al., 2014). Since platelet activation and thrombocytopenia stimulate the inflammatory cascade during sepsis, therapies that target antiplatelets may be effective against it (De Stoppelaar et al., 2014). In inflammatory bowel disease, platelets can activate microvascular endothelial cells to recruit immune cells to sites of inflammation. Like leukocytes, activated platelets adhere to damaged or activated endothelial cells while interacting with other immune cells via P selectin-mediated interaction (De Stoppelaar et al., 2014; Morrell et al., 2014). Thus, wrapping nanoparticle cores with platelet membrane can produce therapeutic systems that target inflammatory diseases.

Nanoparticles cloaked in platelet membrane have been shown to adhere selectively to damaged blood vessels and to bacteria that normally interact with platelets (Hu et al., 2015). In fact, these nanoparticles have been loaded with the antibiotic vancomycin and shown to target the drug to *Staphylococcus aureus* strain 252.

### Nanoparticles coated with stem cell membrane and their anti-inflammatory activity

Stem and precursor cells regulate differentiation and remodeling through paracrine effects, especially their capacity to migrate to damaged tissues, which is important for tissue repair and regeneration (Rennert et al. 2012). Among these cells, bone marrow mesenchymal stem cells (MSCs) from perivascular cells are present in various tissues and organs, including bone marrow, adipose tissue, peripheral blood, lung, brain and skeletal muscle (Dodson et al., 2010). They can self-renew and differentiate, and they can modulate immune processes in inflammatory bowel diseases, arthritis, lung inflammation, allergic encephalitis and respiratory diseases (Karp & Leng Teo, 2009). MSCs express a variety of chemokines and chemokine receptors, which allows them to target sites of inflammation (Chamberlain et al., 2007).

Biomimetic PLGA nanoparticles coated with stem cell membrane have been shown to selectively interact with damaged cardiac cells to protect the remaining myocardium and improve cardiac function in a mouse model of myocardial infarction (Tang et al., 2017). The nanoparticles did not stimulate T cell infiltration in these mice, suggesting good biosafety and biocompatibility.

### Nanoparticles coated with bacterial cell membrane and their anti-inflammatory activity

**Figure 5.**
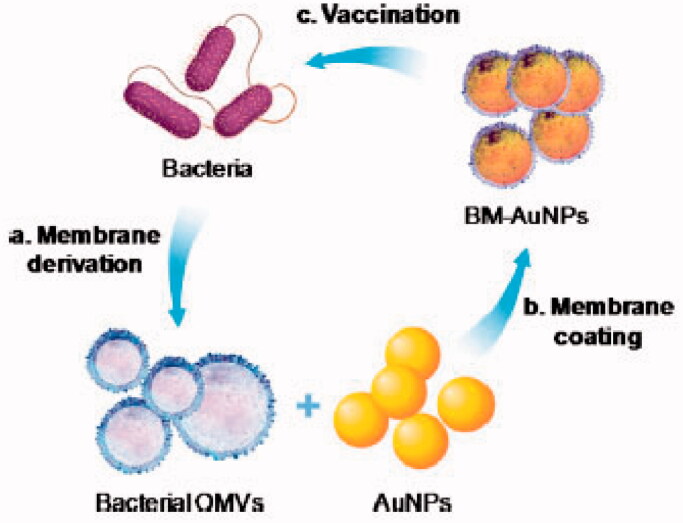
A schematic of how bacterial membrane-coated nanoparticles may modulate antibacterial immunity. AuNPs, gold nanoparticles; BM, bacterial membrane; OMVs, outer membrane vesicles (Gao et al., 2015). Copyright 2015, American Chemical Society.

OMVs from bacteria resemble–to the host–the bacteria from which they came, and the many bacterial surface antigens that they contain can stimulate a protective immune response (Gagliardi, 2017). For example, nanovesicles derived from bacterial protoplasts can serve as adjuvant-free vaccines: such vesicles, when loaded with bacterial antigens, can induce strong antigen-specific humoral and cellular immune responses that provide effective protection against bacterial sepsis (Kim et al., 2015). In one study, gold nanoparticles were wrapped in *Escherichia coli* OMVs to yield nanoparticles with a diameter of 41.9 ± 0.5 nm (Gao et al., 2015) (Figure 5). When injected subcutaneously into mice, these nanoparticles rapidly activated dendritic cells in the lymph nodes. They also effectively elicited bacterium-specific B-cell and T-cell responses in vaccinated animals. These results suggest the potential of designing antibacterial vaccines by coating synthetic nanoparticles with natural bacterial membrane.

### Nanoparticles coated with other types of cell membrane and their anti-inflammatory activity

Endothelial cells are widely present in the body and participate in a number of pathophysiological processes such as inflammation and tumors. Pathogens often act on molecules on the surface of endothelial cell membranes. For example, polymeric nanoparticles were loaded with the antibiotic clarithromycin and coated with membrane from gastric epithelial cells, allowing them to adhere to *Helicobacter pylori* (Angsantikul et al., 2018). These nanoparticles were more effective than free antibiotic or naked nanoparticles against *Helicobacter pylori* infection in mice.

**Figure 6.**
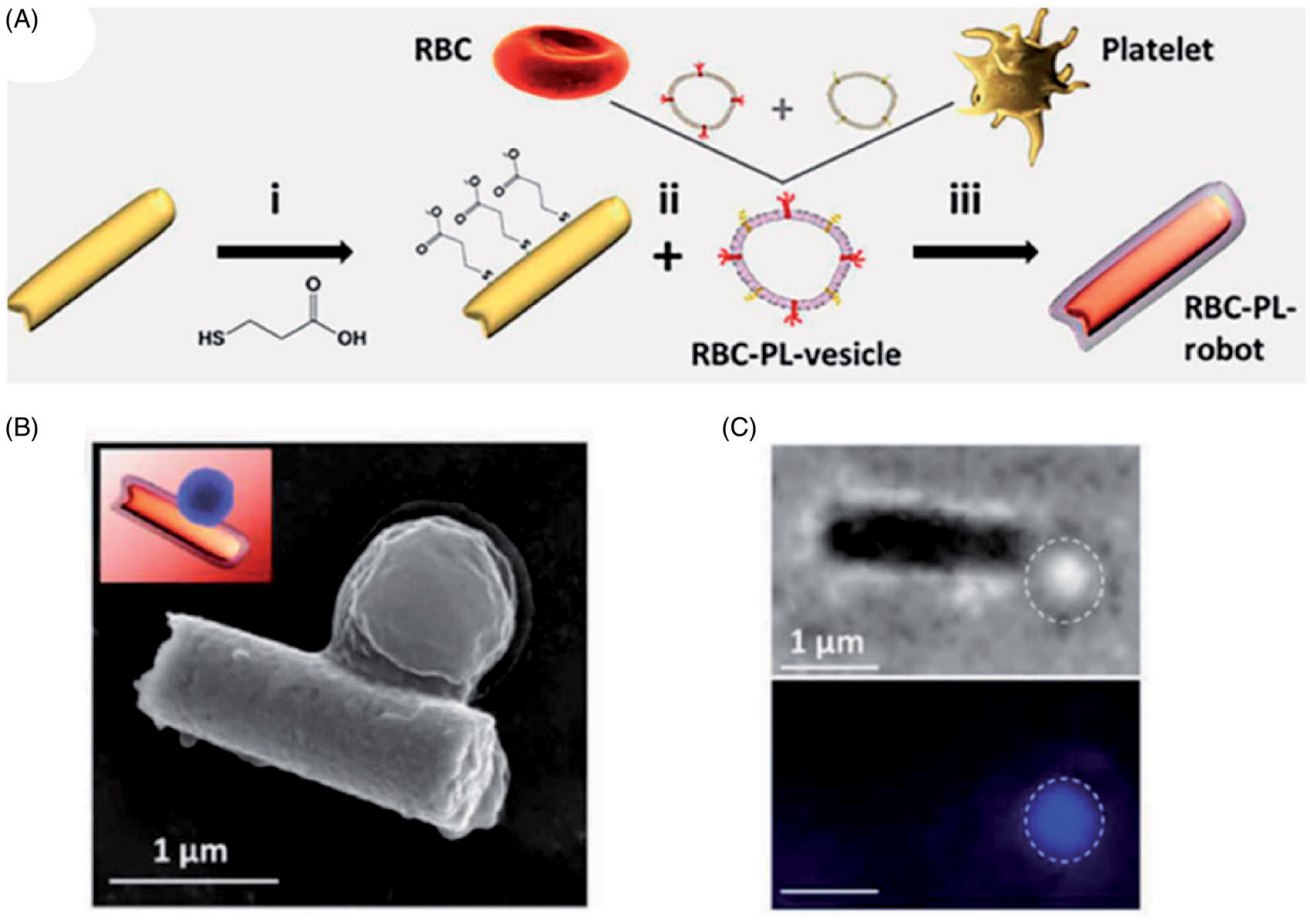
(A) Preparation of a red blood cell (RBC)-platelet (PL)-robot. (B) SEM image of *Staphylococcus aureus* attached to the RBC-PL-robot. (C) Microscope images showing the binding of *Staphylococcus aureus* to an RBC-PL-robot. The *upper* panel shows a brightfield image, and the *lower* panel shows a fluorescence image of a DAPI-stained bacterium (Esteban-Fernández de Ávila et al., 2018). Copyright 2018, American Association for the Advancement of Science.

Researchers have broadened the possible characteristics and functions of membrane-camouflaged nanoparticles by fuzing membranes from multiple types of source cells. For example, wrapping gold nanowires in membranes mixed from RBCs and platelets generated nanoparticles that bound to *Staphylococcus aureus,* which normally bind to platelets, and that neutralized pore-forming toxins such as α-toxin (Esteban-Fernández de Ávila et al., 2018) (Figure 6). It may be possible to tailor nanoparticles to various types of disease by hybridizing the appropriate membranes.

## Conclusions and perspectives

Cell membrane-camouflaged biomimetic nanoparticles combine the intrinsic properties of the cells that served as the membrane source, with the functional versatility of the nanomaterial in the core. This provides new opportunities for prolonging time in circulation, reducing immunogenicity and targeting drug cargo. Better than synthetic drug delivery systems, natural cell membranes can avoid eliciting an immune response and their surface components can interact with receptors on target cells, such as at sites of inflammation or infection. Optimizing the source of the membranes and the composition of the nanoparticle core can lead to biomimetic nanoparticles with unique properties. Applying cell membrane-camouflaged biomimetic nanotechnology to inflammation has shown promise but its implementation in clinical treatments will be challenging because of the complexity and heterogeneity of the inflammatory microenvironment, as well as costly because of the design and production processes involved. By integrating nanotechnology, medicine, materials science, bioengineering and pharmaceutical science, it may be possible to reduce these costs and close the gap between preclinical research and clinical application.
